# The cranial endocast of *Dipnorhynchus sussmilchi* (Sarcopterygii: Dipnoi) and the interrelationships of stem-group lungfishes

**DOI:** 10.7717/peerj.2539

**Published:** 2016-10-20

**Authors:** Alice M. Clement, Tom J. Challands, John A. Long, Per E. Ahlberg

**Affiliations:** 1 School of Biological Sciences, Flinders University, Adelaide, South Australia, Australia; 2 Department of Sciences, Museum Victoria, Melbourne, Victoria, Australia; 3 Department of Organismal Biology, Uppsala Universitet, Uppsala, Sweden; 4 School of Geosciences, University of Edinburgh, Edinburgh, United Kingdom

**Keywords:** Dipnoi, Endocast, Braincase, Palaeoneurology, Devonian, *Dipnorhynchus*, Phylogeny, Microtomography, Cladistic analysis

## Abstract

The first virtual cranial endocast of a lungfish from the Early Devonian, *Dipnorhynchus sussmilchi*, is described. *Dipnorhynchus,* only the fourth Devonian lungfish for which a near complete cranial endocast is known, is a key taxon for clarifying primitive character states within the group. A ventrally-expanded telencephalic cavity is present in the endocast of *Dipnorhynchus* demonstrating that this is the primitive state for “true” Dipnoi. *Dipnorhynchus* also possesses a utricular recess differentiated from the sacculolagenar pouch like that seen in stratigraphically younger lungfish (*Dipterus, Chirodipterus, Rhinodipterus*), but absent from the dipnomorph *Youngolepis*. We do not find separate pineal and para-pineal canals in contrast to a reconstruction from previous authors. We conduct the first phylogenetic analysis of Dipnoi based purely on endocast characters, which supports a basal placement of *Dipnorhynchus* within the dipnoan stem group, in agreement with recent analyses. Our analysis demonstrates the value of endocast characters for inferring phylogenetic relationships.

## Introduction

Lungfish, or dipnoans as they are also known, have origins dating back over 400 million years. Today there are just six extant species, but it was during the Devonian Period that they reached the peak of their success and diversity (Clack, Sharp & Long, 2011). ‘Total-group’ lungfishes are a well-supported monophyletic group, but early dipnoan phylogeny has long been contentious and remains unresolved (Johanson & Ahlberg, 2011). Campbell & Barwick (1990) employed a functional-adaptive method, splitting Palaeozoic lungfishes into three lineages based mainly on dental characters (tooth-plated, dentine-plated and denticulated forms). However, most workers in the field instead utilise cladistic methods; applying the principles of either parsimony (Ahlberg, Smith & Johanson, 2006; Clement, 2012; Marshall, 1986; Miles, 1977; Qiao & Zhu, 2009; Schultze & Marshall, 1993) or Bayesian inference (Friedman, 2007).

The split between the extant lungfish families is thought to have occurred in the Permian (Heinicke, Sander & Hedges, 2009). In general terms this means that ‘crown-group’ lungfishes contains all the living representatives and their last common ancestor (of plausible Permian age), and all of the (both fossil and living) descendants of that ancestor. Thus, ‘stem-group’ lungfish is equivalent to the total group minus the crown group, and contains all of the Devonian lungfishes. The most basal unambiguous member of the lungfish stem group (and sister group to all other lungfishes) is *Diabolepis* from the Lower Devonian of China (Chang, 1995; Chang & Yu, 1984), although some authors disagree with this interpretation (Campbell & Barwick, 2001). The group is thought to have radiated quickly (Lloyd, Wang & Brusatte, 2011), and early Devonian lungfishes are known from deposits across China, Russia, Europe, North America and Australia (Campbell & Barwick, 1986). The Australian fauna from this time is dominated by the robust, short-headed ‘dipnorhynchid’ taxa, typified by *Dipnorhynchus* itself (Etheridge, 1906).

**Figure 1:**
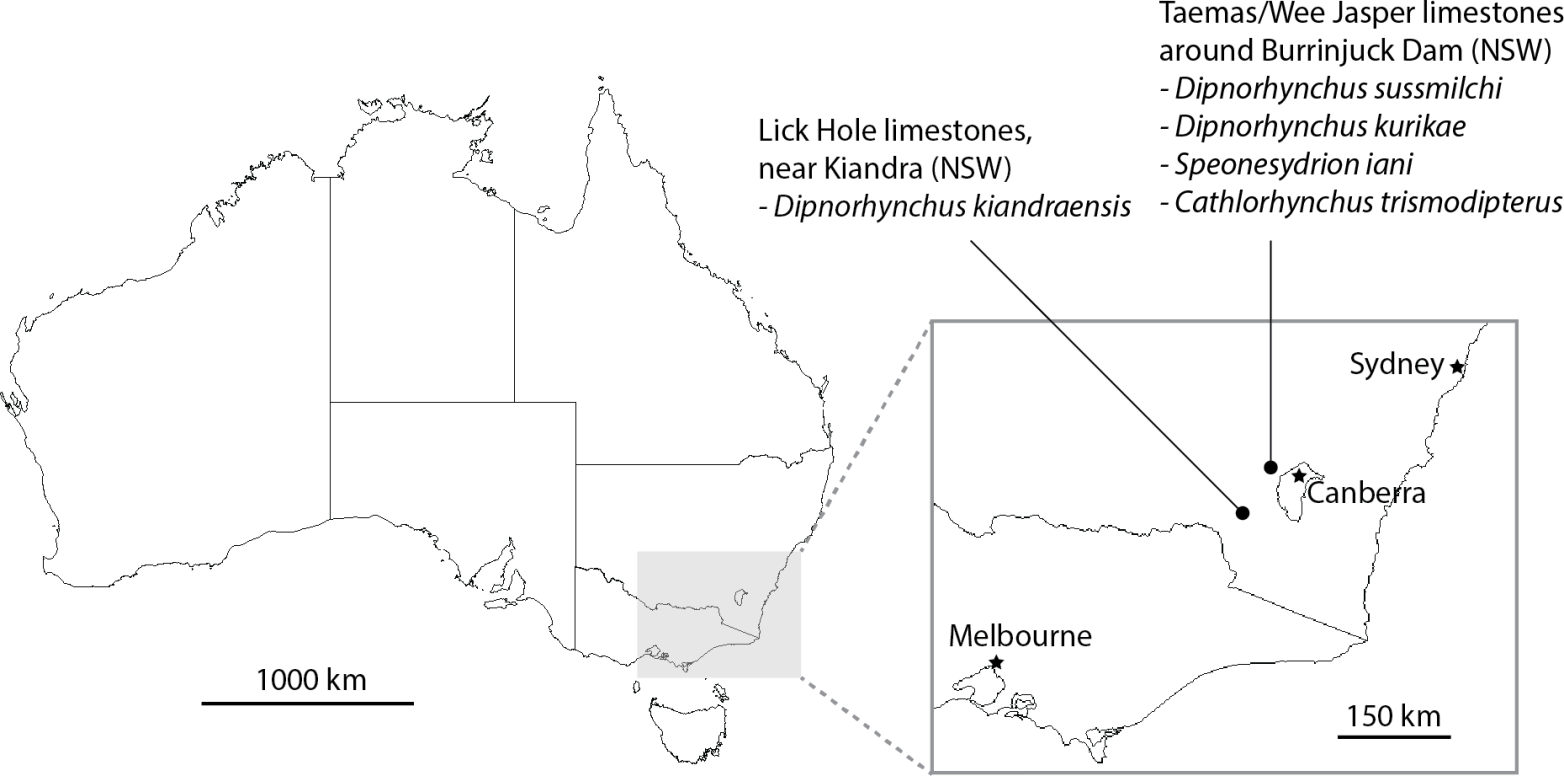
Map showing localities yielding Early Devonian dipnorhynchid taxa in south-eastern Australia.

Material of the Early Devonian genus *Dipnorhynchus* was first described over a century ago, and although the name ‘*Ganorhynchus*’ was originally used (Etheridge, 1906), ‘*Dipnorhynchus*’ was erected two decades later for *D. sussmilchi* (Jaekel, 1927). *Dipnorhynchus sussmilchi* (Campbell & Barwick, 1982a) is known from the Taemas–Wee Jasper limestones that occur around Burrinjuck Dam in New South Wales, Australia (see Fig. 1), and have been dated as Emsian in age (Thomson & Campbell, 1971). Other dipnoan taxa described from the same site include *Dipnorhynchus kurikae* (Campbell & Barwick, 2000), *Speonesydrion iani* (Campbell & Barwick, 1983), and *Cathlorhynchus trismodipterus* (Campbell, Barwick & Senden, 2009). Furthermore, there is an additional *Dipnorhynchus* species known from the Lick Hole Limestones, south of the Taemas–Wee Jasper limestones, *D. kiandrensis* (Campbell & Barwick, 1982b).

The skull roof and associated cranial material of *D. sussmilchi* was first described in some detail (Hills, 1941), and then later elaborated upon (Campbell, 1965; Thomson & Campbell, 1971). However, a thorough investigation of braincase material for the genus did not come until later when Campbell & Barwick (1982a) described the neurocranium and reconstructed the endocranial cavity of *D. sussmilchi*. This was achieved by examining broken specimens to reveal internal anatomy, or by using soft fishing wire to trace the course of small canals. Later, similar treatment for *D. kurikae* ensued (Campbell & Barwick, 2000). Further details regarding the neurocranium and space for the endolymphatic ducts and labyrinth of *D. sussmilchi* shortly followed (Campbell, Barwick & Lindley, 2000).

With the increasing accessibility of modern, non-invasive scanning technology such as synchrotron and micro-computed tomography (µCT), along with more sophisticated software packages for data processing and visualization, the field of palaeoneurology seems to be undergoing an upsurge (Walsh, Luo & Barrett, 2013). Until relatively recently, researchers had to rely upon fortuitous findings of damaged skulls, or resort to destructive techniques (Stensiö, 1963) to examine the internal anatomy of the braincase. These more traditional techniques have been shown to be somewhat limited, especially with respect to fine morphological details (Giles, Rogers & Friedman, 2016). However, today we are quickly increasing the number of taxa for which virtual cranial endocast morphology is known across all vertebrate groups (Balanoff et al., 2015; Falk, 2004; Lu et al., 2012), including fishes such as the sarcopterygian *Powichthys* (Clément & Ahlberg, 2010), the placoderm *Romundina* (Dupret et al., 2014), the galeaspid *Shuyu* (Gai et al., 2011), actinopterygians (Giles & Friedman, 2014; Giles, Rogers & Friedman, 2016; Lu et al., 2016a), and chondrichthyans (Maisey, 2007).

*Chirodipterus wildungensis* from the Upper Devonian of Germany was the first cranial endocast of a lungfish published (Säve-Söderbergh, 1952), although this was drawn from a single damaged specimen and provided only a relatively crude reconstruction. Other examples include a partial endocast of the Late Devonian *Holodipterus* (Pridmore, Campbell & Barwick, 1994), as well as those of *Dipnorhynchus* (Campbell & Barwick, 1982a; Campbell & Barwick, 2000). Although the first virtual lungfish endocasts only came recently, they have greatly enriched our knowledge of the field. Not only are the tomographic methods that produce these endocasts non-destructive, they also provide far more comprehensive information about the cranial cavity, and far superior possibilities for visualization than traditional techniques. Two genera have been investigated by tomography to date: the Late Devonian *Rhinodipterus* from Australia (Clement & Ahlberg, 2014) and *Dipterus* from the Middle Devonian of Scotland (Challands, 2015). Further to this, the brain and endocast of the extant Australian lungfish, *Neoceratodus*, is also known from CT data (Clement et al., 2015), and researchers are developing techniques for reconstructing brains in extinct members (Clement et al., 2016). Not only are cranial endocasts rich sources of morphological data in their own right, they can also give clues as to an animal’s brain structure, sensory abilities and inferred behavior.

We expand on this growing body of work by investigating the cranial endocast of *Dipnorhynchus sussmilchi* from the Early Devonian of Australia as revealed from tomographic data. Our work represents the oldest, and only the fourth lungfish taxon endocast investigated, and is the currently the only example from the Early Devonian. The data from *Dipnorhynchus* contributes to uncovering how the lungfish brain has evolved through time, and also provides valuable data in resolving early dipnoan phylogeny.

## Material & Methods

The *Dipnorhynchus sussmilchi* Etheridge (1906) specimen (ANU 18815) is a well-preserved, acid-prepared complete cranium from the Early Devonian (Emsian) Taemas-Wee Jasper/Burrinjuck limestones of New South Wales, Australia (Fig. 1). The specimen is housed at the Australian National University, Canberra, Australia, and was scanned at the High Resolution X-ray Computed Tomography (µCT) facility of the same location (Sakellariou et al., 2004) with a voxel resolution of 30.4 microns. The ANU µCT facility is based on cone beam geometry and has a detector pixel width of 2048 pixels. The original scan was performed with a focus on snout morphology for another study (Campbell, Barwick & Senden, 2010), and consequently the rear portion of the skull was not captured.

*VGStudio Max*, version 2.2 (Volume Graphics Inc., Germany) was used to achieve three-dimensional segmentation and modeling of the cranial endocast through a combination of manual segmentation and thresholding. The resulting endocast model was smoothed by a factor of three prior to export.

We assembled our character matrix of 20 characters and 10 taxa in *Mesquite 3.01* (see Supplemental Information 1 for full details). Characters 1–13 were taken from previous analyses (Friedman, 2007; Giles et al., 2015); however, 14–20 are new characters identified through the course of this study. The parsimony analysis was conducted using the heuristic search algorithm in *PAUP v4.0b10* (Swofford, 2001) using stepwise addition, 10,000 random addition sequence replicates holding five trees at each step, with tree bisection and reconnection (TBR) enabled, and automatically increasing maxtrees by 100. The Late Devonian coelacanth *Diplocercides* was designated as the outgroup. Bootstrap values were then calculated using 1,000 random replicates of the heuristic search in PAUP, again with TBR enabled for 10 replicates.

## Results

### Description

**Figure 2:**
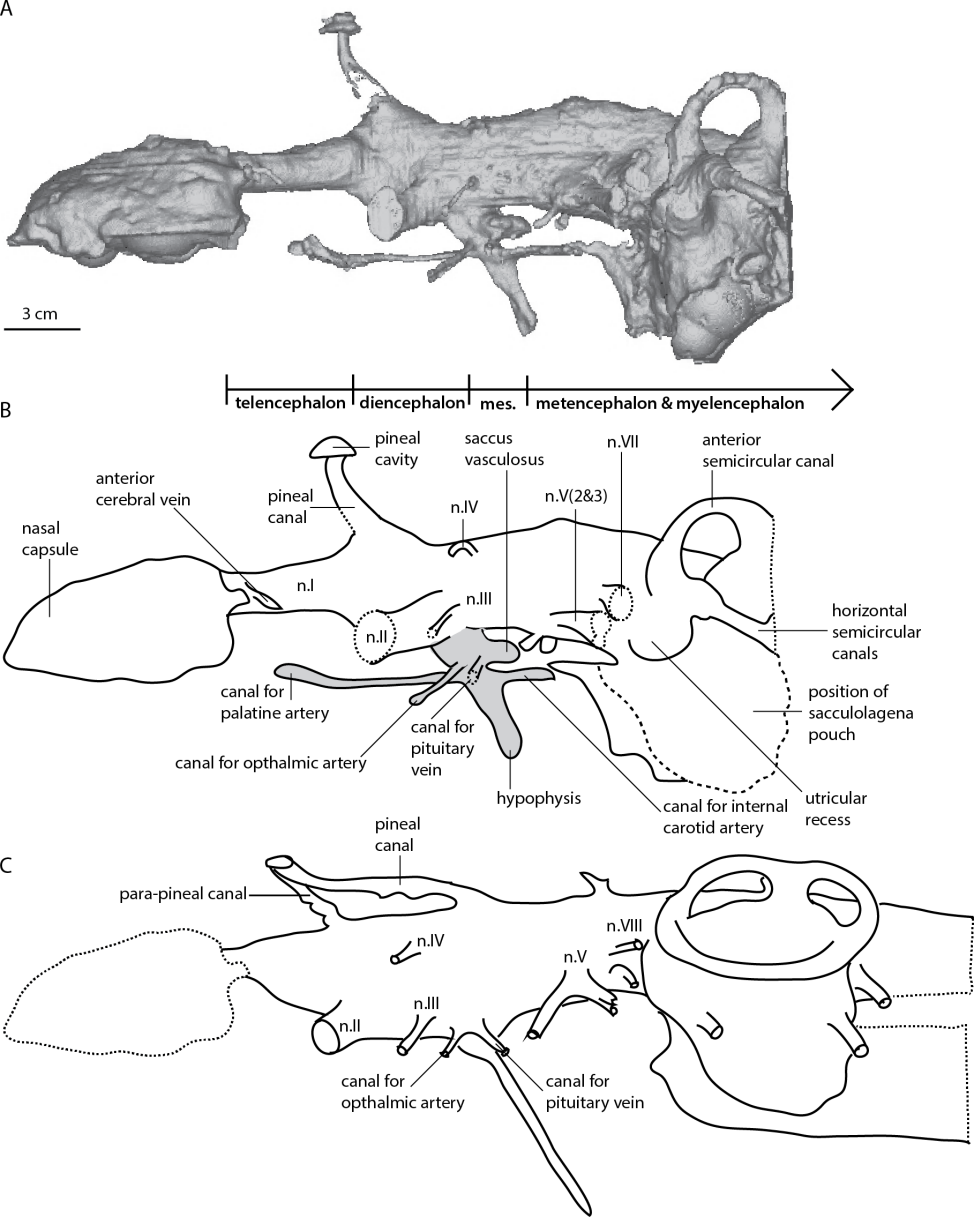
*Dipnorhynchus sussmilchi* cranial endocast in lateral view. (A) virtual reconstruction; (B) schematic illustration of ANU 18815; and (C) reproduction of *D. sussmilchi* endocast from Campbell & Barwick (1982a, Fig. 25A).

**Figure 3:**
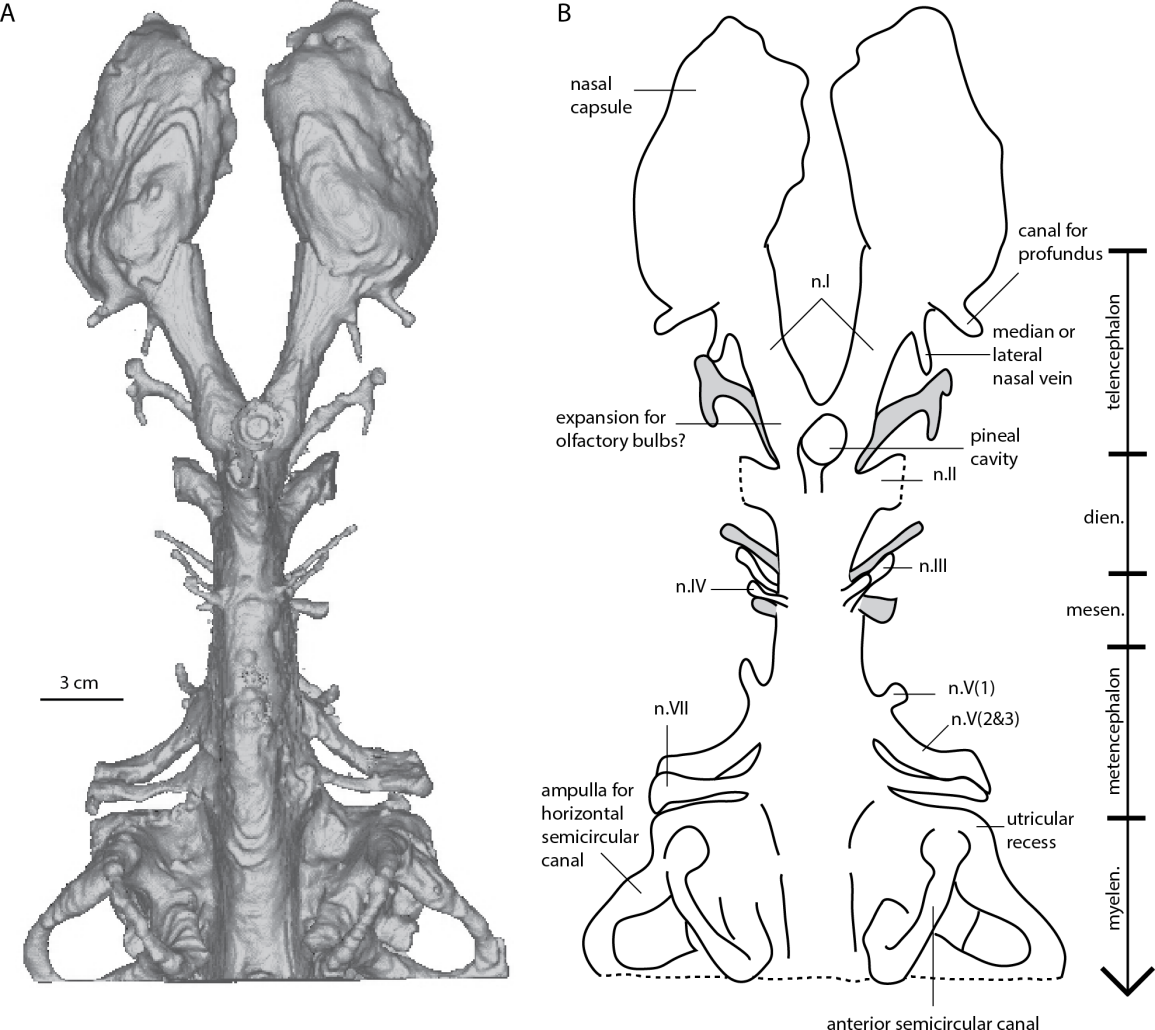
*Dipnorhynchus sussmilchi* cranial endocast in dorsal view. (A) virtual reconstruction; and (B) schematic illustration.

**Figure 4:**
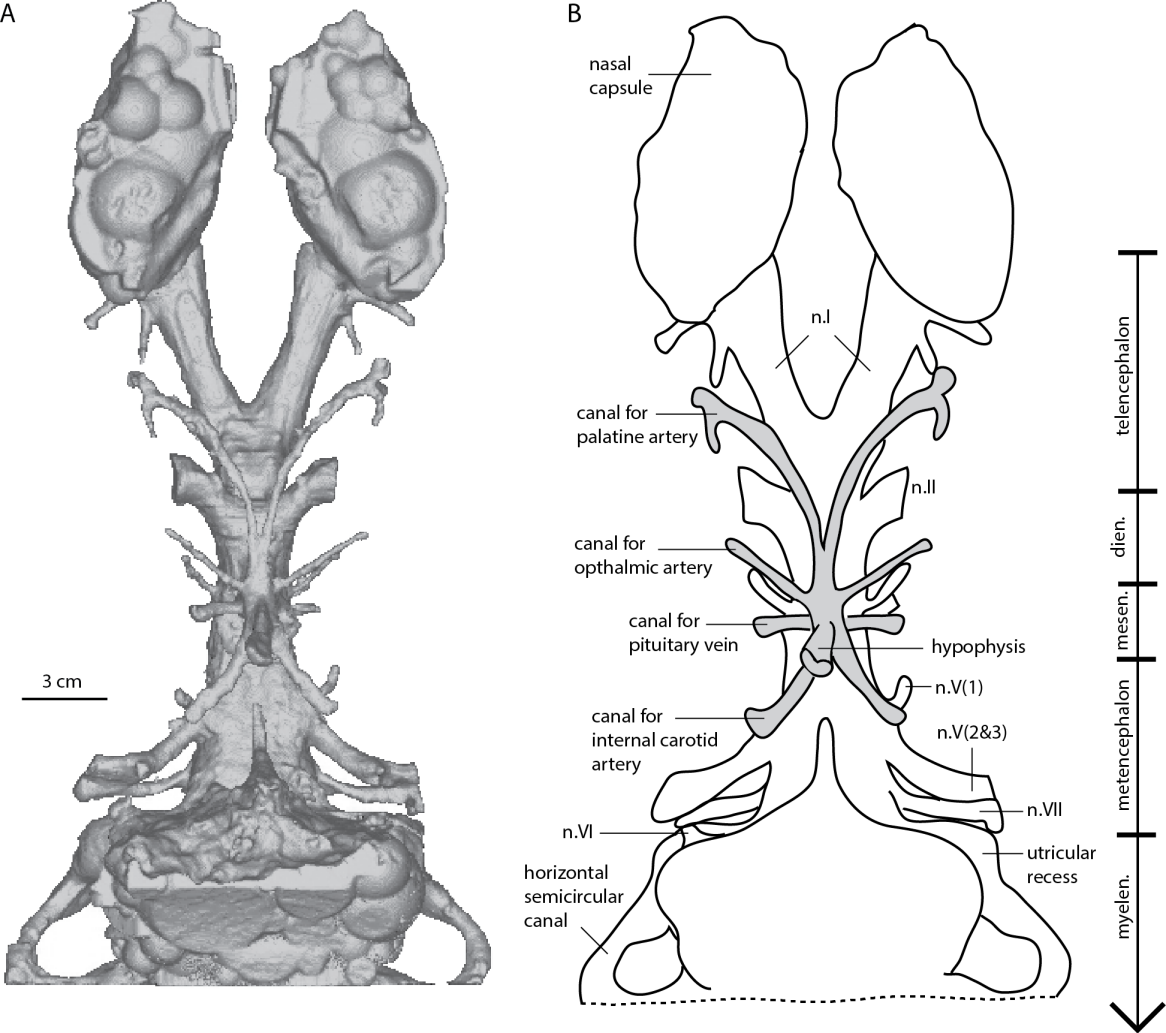
*Dipnorhynchus sussmilchi* cranial endocast in ventral view. (A) virtual reconstruction; and (B) schematic illustration.

**Figure 5:**
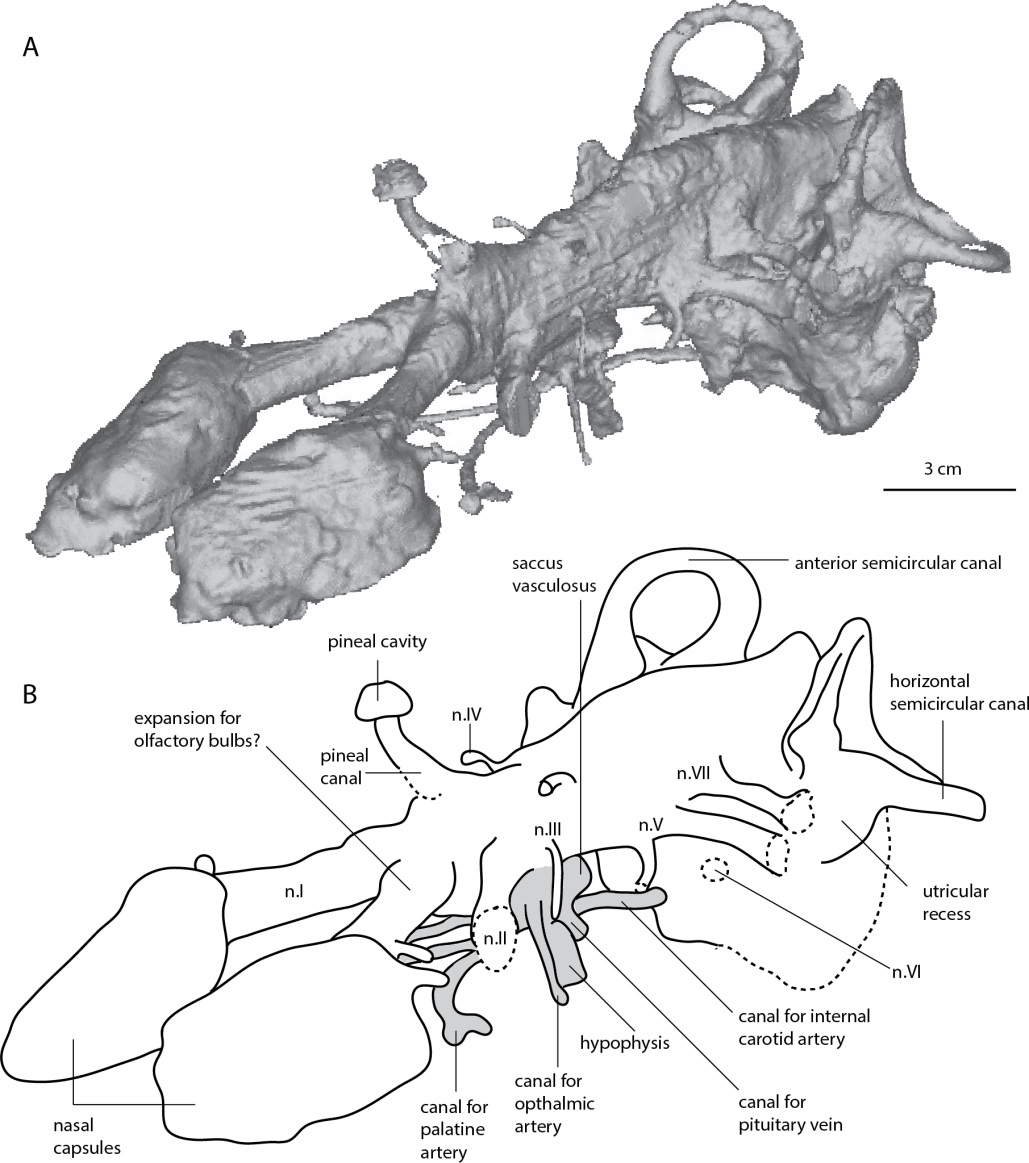
*Dipnorhynchus sussmilchi* cranial endocast in anterodorsolateral view. (A) virtual reconstruction; and (B) schematic illustration.

The skull measures 12 cm in length, and almost 7.5 cm across the quadrates at its widest point (Thomson & Campbell, 1971: Fig. 36). The scan data extends from the tip of the snout to the mid-point of the labyrinth region. Unfortunately the occipital region of the specimen is not included in the scan. Proportionally the nasal capsules are the longest structures of the available endocast being approximately 25% of the total length. The metencephalic and telencephalic regions account for 10–15% endocast length, the diencephalic <10% and the mesencephalic cavity, at ∼5% total length, is the smallest component of the endocast (Figs. 2–5).

#### Nasal capsules and olfactory tracts

The nasal capsules are large, oblong structures with a convex dorsal surface (Fig. 2). The medial edges of the nasal capsules are not parallel, instead they converge anteromedially at an angle of 45 degrees (Figs. 3 and 4). Unlike Thomson & Campbell (1971) we do not recognise an anterior nasal opening for a nostril present, but there is a canal exiting the capsules in their posterolateral corners; this most likely housed the profundus nerve. Challands (2015) noted that in both *Dipnorhynchus sussmilchi* and *Dipnorhynchus kurikae*, the ramus opthalmicus profundus V enters the posterior of the nasal capsule, rather than circumventing it as seen in all other Devonian lungfishes. We see no evidence of threefold sub-divisions as reconstructed for this taxon by Thomson & Campbell (1971, Fig. 29). The canals for the olfactory nerves (n.I) are expanded at their anterior extent where they join the nasal capsules, and a canal likely for either the median or lateral nasal vein, exits in a posterior direction just behind the nasal capsules (Fig. 3). The olfactory tracts are broad and diverge from each other at 45 °, and they are proportionally shorter in *Dipnorhynchys* than in *Dipterus* or *Rhinodipterus* (Challands, 2015; Clement & Ahlberg, 2014). At their posterior extent there are two small rounded expansions visible in each tract that probably housed the olfactory bulbs (Fig. 5).

#### Telencephalic region

The telencephalic region is mostly flat dorsally, reminiscent of the condition seen in *Youngolepis* (Chang, 1982), however there is a strong rise towards the pineal canal posteriorly on the dorsal margin (Fig. 2). There is a small yet distinct expansion of the telencephalic ventral edge, mostly obscured behind the large canals for the optic nerves. However, there is no evidence of a distinctive lateral telencephalic expansion like that originally reconstructed in *Dipnorhynchus* (Campbell & Barwick, 1982a), nor those seen in *Chirodipterus wildungensis* (Säve-Söderbergh, 1952), *Rhinodipterus kimberleyensis* (Clement & Ahlberg, 2014), nor extant lungfishes (Clement et al., 2015; Northcutt, 2011).

#### Diencephalic region

The diencephalic cavity is slightly narrower than the telencephalic region and also about one-third shorter (Figs. 3 and 4). Two wide, cylindrical anterolaterally-directed canals for the optic nerves (n.II) exit the endocast in a ventral position at the anterior boundary of the diencephalic region. *D. sussmilchi* carries a posteroventrally long hypophyseal recess with a number of smaller, well-defined canals entering it (Figs. 2 and 4). The hypophyseal recesses of *Rhinodipterus* and *Dipterus* do not extend as far ventrally (Challands, 2015; Clement & Ahlberg, 2014), however these structures in *Youngolepis* (Chang, 1982) and *Eusthenopteron* (Stensiö, 1963) are of similar proportions to *Dipnorhynchus*. The hypophyseal cavity reconstructed from our scan data differs in a number of aspects from that of Campbell & Barwick (1982a, Fig. 25). As our reconstruction is based directly on a scan of the cavity, whereas Campbell and Barwick’s reconstruction was based on inferences from partly visible structures, we believe the differences reflect the limitations of the latter technique. Dorsal to the hypophysis lies a short, rounded saccus vasculosus oriented posteriorly underneath the cranial cavity (Fig. 2), similar to those seen in actinopterygians (Giles et al., 2015; Giles & Friedman, 2014) or chondrichthyans (Maisey, 2007). There are a number of paired canals exiting the hypophysis (Figs. 2, 4 and 5); the most anterior of these extend far anterior, as far as the optic nerve canals before extending outwards laterally and likely housed the palatine arteries. The posteriorly-directed canals diverge outwards towards the trigeminal nerves and probably contained the internal carotid arteries. It is interesting to note that the canal for the internal carotid (Fig. 4) does not appear to bifurcate for a branch for the pseudobranchial artery like seen in *Dipterus* (Fig. 10B, Challands, 2015) and other stratigraphically younger lungfish. Slightly dorsal to this canal are two small canals interpreted as housing the pituitary veins. Slightly anterior to these canals are two further canals directed in an anterolateral direction that probably housed the ophthalmic arteries. Along the dorsal edge of the diencephalic region lies a broad eminence from which the pineal canal leads upwards towards the circular pineal gland in the skull roof (Fig. 2). The pineal gland is situated further anterior than that originally drawn for *Dipnorhynchus* (Campbell & Barwick, 1982a). We find no obvious evidence of a parapineal gland (*contra* Campbell & Barwick, 1982a), however there are a number of miniscule canals leaving from along the midline towards the skull roof dorsally in this area.

#### Mesencephalon

The mesencephalic cavity is the shortest region of the endocast and is as narrow as the diencephalic cavity. On the lateral face of the endocast are two small paired canals exiting in anterolateral directions (Fig. 2); the ventrally lower one would have housed the oculomotor nerve (n.III), and the dorsally higher one the trochlear nerve (n.IV). The ventral and dorsal edges of the mesencephalic cavity are fairly flat, about twice as high as the telencephalic region.

#### Metencephalic and Myelencephalic cavities

The metencephalic region extends from the bifurcating canals for the trigeminal nerves (n.V) to a poorly defined region posterior to the canals for the auditory nerves (n.VIII), although a distinct boundary cannot be determined. The canal for the ophthalmic nerve (n.V_1_) extends anterolaterally, while the combined canal for maxillary and mandibular nerves (n.V_2&3_) is broader and extends in a posterolateral direction. Slightly posterodorsal to this, the canal for the facial nerve (n. VII) extends laterally (Figs. 2 and 5). There is a further canal anterior to and slightly ventral to the utricular recess that could have housed the abducens (n. VI) nerve (Figs. 4 and 5). Unlike Campbell and Barwick, we have not been able to identify the canals for the auditory (n. VIII) nerves from the data (Fig. 2). The anterior portion of the metencephalic region is of similar width to the preceding mesencephalon, thought it widens laterally slightly towards its posterior extent. The ventral margin is straight but the dorsal surface is gently curved to form a convex margin forming the deepest brain region (Fig. 2). There are no prominent supraotic cavities like those seen in *Rhinodipterus* present (Clement & Ahlberg, 2014). Very little can be said concerning the myelencephalic region as most of this is missing from the scan, however it appears to have a slightly lower dorsal margin than the metencephalic region but is of similar width.

#### Labyrinth region

Although the labyrinth region is incomplete, we can still observe a number of salient features. The anterior semicircular canals stand much higher than the dorsal extent of the hindbrain and *Dipnorhynchus* presumably possessed a relatively tall superior sinus (Fig. 2). There is a large ampulla on the anterior semicircular canal, and although its full extent cannot be determined, the sacculagenar pouch appears to have at least been long. The utricular recess is only moderately expanded. This is in contrast with more derived lungfishes such as *Rhinodipterus* (Clement & Ahlberg, 2014), *Chirodipterus* (Säve-Söderbergh, 1952), and *Dipterus* (Challands, 2015), although we note it is more expanded than the reconstruction by Campbell & Barwick (1982a, Fig. 25).

### Phylogenetic analysis

The phylogenetic analysis implemented herein focuses solely on characters identifiable from cranial endocasts, and is far from comprehensive. The approach of Friedman (2007) in using the whole neurocranial complex can include a greater wealth of data than our analysis. However great care must be taken so as not to score the same character twice, once described from the neurocranium and once as an endocast feature. Indeed, the results of the phylogenetic analysis are preliminary, but it is our hope that with increasingly accessible scanning technology and a greater number of specimens examined our character matrix will demonstrate the efficacy of endocast characters in their own right and serve as a framework for future analyses and new data, and allow workers to infer phylogeny from endocasts in cases where associated neurocranial data is not adequately provided.

**Figure 6:**
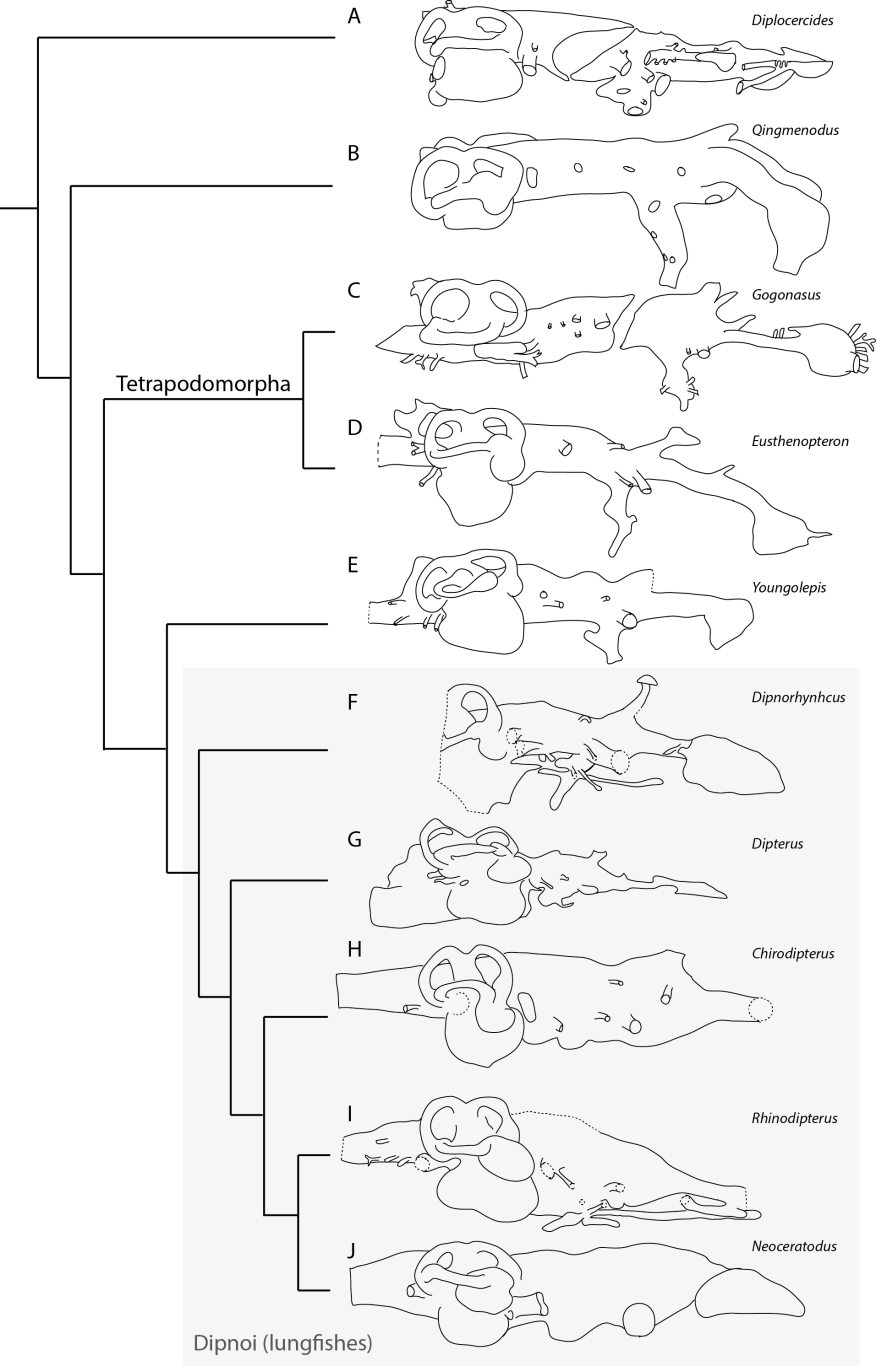
Phylogenetic relationships of selected sarcopterygians as interpreted from cranial endocast morphology. (A) the Late Devonian coelacanth *Diplocercides kayseri* (from Stensiö, 1963, Fig. 45); (B) the Early Devonian onychodont *Qingmenodus yui* (from Lu et al., 2016b, Fig. 2); the Late Devonian tetrapodomorphs (C) *Gogonasus andrewsae* (from Holland, 2014, Figs. 22 and 23); (D) *Eusthenopteron foordi* (Stensiö, 1963, Fig. 50); (E) the Early Devonian dipnomorph *Youngolepis praecursor* (from Chang, 1982, Fig. 19); (F) the Early Devonian dipnoan *Dipnorhynchus sussmilchi* (ANU 18815); (G) the Middle Devonian dipnoan *Dipterus valenciennesi* (from Challands, 2015, Fig. 9); the Late Devonian dipnoans (H) *Chirodipterus wildungensis* (from Säve-Söderbergh, 1952, Fig. 9); (I) *Rhinodipterus kimberleyensis* (WAM 09.6.149); and (J) the extant Australian lungfish *Neoceratodus forsteri* (from Clement et al., 2015, Fig. 6).

The comparative endocast data used in our analysis was taken from the literature (Challands, 2015; Chang, 1982; Clement & Ahlberg, 2014; Clement et al., 2015; Holland, 2014; Lu et al., 2016b; Säve-Söderbergh, 1952; Stensiö, 1963). Although only small, the results of our analysis (Fig. 6) focusing on cranial endocast characters mostly reflect the hypotheses of relationships seen in other recent phylogenetic analyses of lungfishes and other Devonian sarcopterygians (Challands, 2015; Clement, 2012; Lu et al., 2016b; Qiao & Zhu, 2009).

The maximum parsimony analysis produced a strict consensus tree with a score of 34 steps, and a consistency index (CI) and retention index (RI) of 0.68, homoplasy index (HI) of 0.32, and a rescaled consistency index (RCI) of 0.46. *Qingmenodus* is the most basal taxon above the outgroup *Diplocercides*. The tetrapodomorphs *Gogonasus* and *Eusthenopteron* form a clade as sister group to the lungfish total group (Dipnomorpha). *Youngolepis* is the most basal taxon in the Dipnomorpha, with *Dipnorhynchus* the most basal of the Dipnoi. *Dipterus, Chirodipterus* and *Rhinodipterus* are more derived occupying successive branches, with *Neoceratodus* comprising a crownward position (Fig. 6).

The results of the phylogenetic analysis do support the use of endocast characters in analyses, either in isolation or in conjunction with other morphological characters. Although virtual palaeoneurology is still its infancy, especially with respect to fishes, cranial endocasts show great potential with which to support hypotheses of phylogeny.

## Discussion

### (a) The *Dipnorhynchus sussmilchi* endocast

The first reconstruction of the cranial endocast of *Dipnorhynchus* was drawn directly from broken specimens and inferring internal morphology without the aid of scanning technology (Campbell & Barwick, 1982a), similar to the method used for *Chirodipterus wildungensis* (Säve-Söderbergh, 1952). In comparison with that reconstruction, we largely agree with most characters including the placement of the cranial canals and general proportions of brain regions. However the most striking point of difference is that there is no large recess for a separate para-pineal canal visible in our scan data (*contra* Campbell & Barwick, 1982a, Fig. 25). There is a large, obvious space for the pineal canal (Fig. 2B), but our specimen of *Dipnorhynchus sussmilchi* appears to lack any separate para-pineal canal. Furthermore, the position of the pineal gland is placed further anteriorly in our reconstruction compared to that of Campbell & Barwick (1982a), at the level of the optic nerve canals rather than at the level of n. IV; this is more in line with the generalized gnathostome condition. There are, however, a number of minute canals exiting the cranial cavity dorsally in this dorsal region of the forebrain that may have been related to the pineal organ.

Another significant difference is the lack of any noticeable lateral expansion in the telencephalic region in contrast to that shown by Campbell & Barwick (1982a). Instead the narrow forebrain appears similar to that of the Early Devonian dipnomorph, *Youngolepis* (Chang, 1982, Fig. 19) in this respect though the presence of a small ventral expansion in *Dipnorhynchus* is more reminiscent of the condition seen in *Dipterus* (Challands, 2015). Similarly, Campbell & Barwick (1982b) did not reconstruct an utricular recess outwardly differentiated from the sacculolagenar pouch. However, we find differentiated sacculolagenar–utricular recesses. The condition is more similar to that reconstructed for *Dipnorhynchus kurikae* by the same authors (Campbell & Barwick, 2000, Fig. 4).

New characters that can be identified in our scan data include the size and shape of the nasal capsules, the position of the nasal vein, as well as details concerning the canals exiting the hypophysis. We are able to distinguish and trace the course of the canals for the palatine, ophthalmic and internal carotid arteries, as well as the canal for the pituitary vein (Figs. 3 and 4).

### (b) Comparison with other sarcopterygians

In Fig. 6 the updated endocast of *Dipnorhynchus* is compared with that of other Devonian lungfishes from which a complete cranial endocast is known, as well as the Early Devonian dipnomorph, *Youngolepis,* two tetrapodomorph taxa (*Gogonasus* and *Eusthenopteron*), the onychodont *Qingmenodus,* and *Diplocercides* the coelacanth. In the forebrain, the slight ventral expansion of the telencephalic region in *Dipnorhynchus sussmilchi* contrasts with the more pronounced expansion in the stratigraphically younger *Chirodipterus wildungensis* and *Rhinodipterus kimberleyensis*. This trend and its implications have already been discussed (Clement & Ahlberg, 2014). It was proposed that this trend of increasing size of the telencephalic region might correlate with an increased reliance on olfaction in lungfishes over time. However, it may also reflect an increased capacity to navigate environmentally complex ecosystems or social systems, as seen in chondrichthyans (Yopak et al., 2007). Two slight bulges at the base of the olfactory nerves (see Fig. 5) suggest that the olfactory bulbs were sessile rather than pedunculate. Relatedly, we believe that the identification of the olfactory bulbs in *Rhinodipterus* may have been originally overlooked; a slight bulge in telencephalic region just posterior of the olfactory canals could represent these (Clement & Ahlberg, 2014, Fig. 2) as is the condition interpreted in *Dipterus* also (Challands, 2015).

Posterior to the pineal recess in *Dipnorhynchus* and *Dipterus* (but apparently lacking in *Chirodipterus*) lies a small bulge on the dorsal surface of the hindbrain region of the endocast. Challands (2015, Fig. 9) tentatively identified this as the space for the optic lobes in *Dipterus*. Campbell & Barwick (1982a); Campbell & Barwick (1982b) reconstructed a single dorsally oriented canal in this region, but we again could not locate such a canal in the tomographic data. Unfortunately this region of the skull was damaged in *Rhinodipterus* so its morphology cannot be determined for this taxon.

As previously discussed (Challands, 2015; Clement & Ahlberg, 2014), Devonian and later lungfishes show a trend of increasing size of the utricular recess relative to the sacculolagena, and that of *Dipnorhynchus* remains small and relatively undifferentiated in comparison to later taxa. Not surprisingly, it closely resembles that of *Dipnorhynchus kurikae* (Campbell & Barwick, 2000, Fig. 5). Moreover, *Dipterus* and *Rhinodipterus* both possess a small notch demarcating the lagenar and saccular portions of the labyrinth region, while this is absent in *Chirodipterus* and extant lungfishes (Clement & Ahlberg, 2014, Fig. 4). All of the lungfish, as well as *Youngolepis, Gogonasus* and *Eusthenopteron* possess a high superior sinus that extends dorsally above the endocranial roof.

Overall the updated endocast of *Dipnorhynchus* closely resembles that of *Youngolepis* and *Diplocercides* in possessing a ventrally-extensive hypophyseal recess, and in lacking any telencephalic lateral expansion. However the emergence of a differentiated utricular recess, ventral expansion of the telencephalon (albeit only slight) and a combined anterodorsally-oriented para-pineal gland resembles those of stratigraphically younger lungfish. These latter features lend support to the primitive placement of *Dipnorhynchus* within the Dipnoi more basally than other lungfish for which the endocranial anatomy is known.

### (c) Evolutionary significance

The cranial endocast of *Dipnorhynchus* exhibits conditions typical for primitive sarcopterygians. A small utricular recess is shared with *Youngolepis, Eusthenopteron, Diplocercides* and *Qingmenodus* implying that the expansion of the utricular recess is a derived condition (synapomorphy) within the dipnoans. Also, unlike more derived lungfishes (*Chirodipterus, Rhinodipterus, Neoceratodus), Dipnorhynchus* retains a buccohypophyseal opening and lacks separate foramina for the internal carotid artery and efferent pseudobranchial arteries. On the other hand, *Dipnorhynchus* demonstrates the derived condition in lacking an intracranial joint, and shows a sinus superior that obviously extends above the roof of the rhombencephalon; a character first observed in early actinopterygians (Giles, Rogers & Friedman, 2016), and the tetrapodomorphs *Eusthenopteron* and *Gogonasus,* but absent in *Qingmenodus* and *Diplocercides* (Fig. 6).

Of further note is the proportion of the hindbrain, the rhombencephalon, relative to other Devonian sarcopterygians. *Dipnorhynchus* and all subsequent dipnoans demonstrate a shortening of the distance between the hypophyseal recess and rhombencephalon, a state more akin to basal actinopterygians such as *Mimipiscis* (Giles & Friedman, 2014). As such, this character appears to be a synapomorphy for the Dipnoi, convergent with the condition in Actinopterygii (Giles, Rogers & Friedman, 2016), the shortening likely related to the loss of the intracranial joint (also seen in the dipnomorph *Youngolepis*). Lu et al. (2016b) noted that the rhombencephalon in Devonian sarcopterygians is generally well-developed to the anterior, with the facial nerve (n.VII) being located well behind the trigeminal complex and anterior to the labyrinth. They tentatively attributed this expansion to an increased demand for functional sensitivity processed in this region of the brain (e.g., motor control in the pons and cerebellum), which if correct, has reverted to the primitive actinopterygian condition in the first appearance of the Dipnoi. Other morphological correlates with well-developed facial functional sensitivity are, however, still present in the Dipnoi such as in *Dipterus* where Challands (2015) noted the extensive innervation in the rostral region by the facial nerve (n.VII) as well as the lateral line by the same nerve complex. Such patterns illustrate the complexities involved in simply attributing endocast volume to increased functional processing for a certain region of the brain. Inferring functional evolutionary trajectories from endocast volumes, especially where demarcation between regions is to a certain extent subjective, is, at best, speculative unless the nervous and functional systems are considered as a whole.

## Concluding Remarks

Here we present the oldest lungfish cranial endocast known, that of *Dipnorhynchus sussmilchi*, from the Early Devonian of Australia, as reconstructed from tomographic data. The virtual endocast presented herein largely confirms the previous depiction by Campbell & Barwick (1982a) however there exist a number of notable differences. These include the lack of a separate para-pineal gland, and similarly the lack of any lateral expansion of the telencephalon. However the presence of a small, but differentiated utricular recess, and new details of the canals exiting the ventrally-extensive hypophysis are revealed.

As only the fourth Devonian lungfish endocast known, *Dipnorhynchus* represents a significant contribution to the field of vertebrate palaeoneurology. However, to be able to draw more useful conclusions concerning dipnoan phylogeny or function we must continue to expand our knowledge base. Neurocrania (and consequently their associated cranial endocasts) are morphologically complex and phylogenetically informative structures, as has already been well illustrated for lungfishes (Friedman, 2007). We present the first character matrix based solely on endocast characters for lungfishes. The analysis supports a basal placement of *Dipnorhynchus* within the dipnoan stem group in agreement with other recent phylogenetic analyses demonstrating the robustness of endocasts alone in creating instructive phylogenetic hypotheses.

##  Supplemental Information

10.7717/peerj.2539/supp-1 Supplemental Information 1 Supplementary Information **Part A. Anatomical abbreviations**
**Part B. Phylogenetic Analysis**
(1) List of Taxa
(2) Character List
**Part C. Supplementary References**

10.7717/peerj.2539/supp-2 Figure S1 Two slices through the CT scan data of *Dipnorhynchus sussmilchi* showing quality of scans, and with certain features labelled

## Additional Information and Declarations

### Data Availability

The following information was supplied regarding data availability: The raw data files, including the CT scans, are available at the Dryad Digital Repository: DOI: 10.5061/dryad.22dr1.
