# Supplementary material for: The cranial endocast of Dipnorhynchus sussmilchi (Sarcopterygii: Dipnoi) and the interrelationships of stem-group lungfishes
Source: PeerJ. 2016 Oct 20;4:e2539. doi: 10.7717/peerj.2539 (PMC5075708; doi:10.7717/peerj.2539)
Supplement: Supplemental Information 1 — Part A. Anatomical abbreviations Part B. Phylogenetic Analysis (1) List of Taxa (2) Character List Part C. Supplementary References [file peerj-04-2539-s001.docx]

**SUPPLEMENTARY INFORMATION**

**TABLE OF CONTENTS**

**Part A. Anatomical abbreviations**  **page 2**

**Part B. Phylogenetic Analysis**  **page 3**

1) List of Taxa page 3

2) Character List page 3

**Part C. Supplementary References**  **page 7**

**Part A. Anatomical Abbreviations**

Dien. Diencephalon

Mesen. Mesencephalon

Myelen. Myelencephalon

n.I canal for olfactory nerve I

n.II canal for optic nerve II

n.III canal for oculomotor nerve III

n.IV canal for trochlear nerve IV

n.V(1) canal for profundus (trigeminal nerve V)

n.V(2&3) canal for combined maxillary & mandibular nerves

n.VI canal for abducens nerve VI

n.VIII canal for auditory nerve VIII

**Part B. Phylogenetic Analysis**

**1) List of Taxa**

| **Taxon** | **Literature** |
| --- | --- |
| *Chirodipterus wildungensis* | ([Säve-Söderbergh 1952](#_ENREF_12)) |
| *Diplocercides kayseri* | ([Stensiö 1963](#_ENREF_13)) |
| *Dipnorhynchus sussmilchi* | ([Campbell & Barwick 1982](#_ENREF_1)); ANU 18815 |
| *Dipterus valenciennesi* | ([Challands 2015](#_ENREF_2)) |
| *Eusthenopteron foordi* | ([Stensiö 1963](#_ENREF_13)) |
| *Gogonasus andrewsae* | ([Holland 2014](#_ENREF_8)) |
| *Neoceratodus forsteri* | ([Clement et al. 2015](#_ENREF_5)) |
| *Qingmenodus yui* | ([Lu et al. 2016](#_ENREF_10)) |
| *Rhinodipterus kimberleyensis* | ([Clement & Ahlberg 2014](#_ENREF_4)) |
| *Youngolepis praecursor* | ([Chang 1982](#_ENREF_3)) |

**2) Character List**

List of characters used in phylogenetic analysis of *Dipnorhynchus sussmilchi* (ANU 18815), cranial endocast only. Characters 1–13 are those from Friedman ([Friedman 2007](#_ENREF_6)) and Giles et al. ([Giles et al. 2015](#_ENREF_7)) pertaining to endocast morphology and applicable to lungfishes. The following characters, 14–20 are new. Ordered multistate characters are indicated with an asterisk (*), unknown characters are marked as’?’, those non–applicable are marked as ‘–‘.

**1.** **Nasal capsule ventral face***

*(Friedman 2007, #46)*

0. wholly ossified

1. perforated

2. unossified

**2. Ossified rostral tubuli developed in ethmoid region**

*(Friedman 2007, #52)*

0. no

1. yes

**3.** **Buccohypophysial canal pierces parasphenoid**

*(Friedman 2007, #67;* Giles et al. 2015 *#124)*

0. yes

1. no

**4.** **Hypophyseal chamber orientation**

*(Giles et al. 2015 #124)*

0. posteroventrally

1. ventrally or anteroventrally

**5.** **Separate foramina for internal carotid artery and efferent pseudobranchial artery**

*(Friedman 2007, #28)*

0. no

1. yes

**6.** **Pineal foramen**

*(Friedman 2007, #39; Giles et al. 2015, #15)*

0. open/present

1. closed/absent

**7.** **Pineal eminence**

*(Friedman 2007, #40)*

0. absent

1. present

**8.** **Pineal/parapineal in separate ossified canals**

*(Friedman 2007, #38)*

0. yes

1. no

**9.** **Optic lobes**

(*Giles et al. 2015, #129)*

0. narrower than cerebellum

1. equal or greater width than cerebellum

**10.** **Endoskeletal intracranial joint**

*(Friedman 2007, #1; Giles et al. 2015, #15)*

0. present

1. absent

**11.** **Notochord extends to or beyond level of n.V**

*(Friedman 2007, #25)*

0. yes

1. no

**12.** **Ampulla expansion in posterior semicircular canal**

*(Friedman 2007, #21)*

0. large

1. reduced or absent

**13.** **Crus commune of anterior and posterior semicircular canals**

*(Giles et al. 2015, #134)*

0. extends dorsal to endocranial roof

1. does not reach level of endocranial roof

**New characters**

**14.** **Utricular recess***

*Clement and Ahlberg (2014) made note of the gradual expansion of the utriculus within the Dipnoi over time. The dipnomorph* Youngolepis *shows no differentiation of the utriculus from the sacculolagenar pouch, whereas Late Devonian lungfishes for which the labyrinth morphology is known (‘*Chirodipterus’ australis, Chirodipterus wildungensis, Griphognathus whitei, Rhinodipterus kimberleyensis*) show an obvious protrusion. Within the extant taxa, the Lepidosirenid taxa are more derived with respect to this character in having the utriculus significantly extended posteriorly and ventrally as well as anteriorly.*

0. indistinct or only a very slight protrusion

1. (obviously) differentiated from sacculolagenar pouch

2. highly differentiated and anteriorly extended

**15.** **Telencephalon expansion**

*This is another character noted by Clement and Ahlberg (2014) who discussed the expansion of the telencephalic region of the cranial endocast in lungfishes over time. Compared to the flat ventral margin seen in* Youngolepis, *even Early Devonian lungfishes show an expansion of the telencephalic region (i.e.* Dipnorhynchus*). This expansion is comparatively larger in* Chirodipterus wildungensis *and in* Rhinodipterus *we begin to see lateral expansion also.*

1. Narrow and with a flat ventral margin
2. Small ventral expansion
3. Large ventral and lateral expansion

**16.** **Olfactory bulbs**

*Among sarcopterygian fishes, pedunculate olfactory bulbs are known in the extant coelacanth (*Latimeria*) and the Australian lungfish (*Neoceratodus*). Whereas the Lepidosirenid lungfishes (*Protopterus *and* Lepidosiren*) appear to show the apomorphic condition, most likely the result of paedomorphosis, as suggested by Northcutt (*[*1986*](#_ENREF_11)*) and supported by recent work on* Neoceratodus *by Clement et al. (*[*2015*](#_ENREF_5)*).*

1. sessile
2. pedunculate

**17. Sacculolagenar pouch**

*The lagena is considered the third otolith endorgan in vertebrates (after the utriculus and sacculus) and is present in all nonmammalian vertebrates (*[*Khorevin 2008*](#_ENREF_9)*). Coelacanths, including* Diplocercides*, are known to have separate pouches for the sacculus and lagena, whereas all lungfishes, as well as tetrapodomorph taxa typically have a common pouch.*

0. separate

1. common pouch

**18. Angle between anterior and posterior semi–circular canals**

*3D geometric morphometrics of the angle between anterior and posterior semi–circular canals in living and fossil Dipnoi demonstrates a statistically– valid trend in decrease in this angle in more derived lungfish taxa (Henderson, unpublished BSc thesis, University of Edinburgh, 2016).*

0. High angle (>100^o^)

1. Low angle (<99^o^)

**19. Median dorsal canals posterior to the pineal–parapineal recess**

*A number of small canals are present on the dorsal surface of the endocast in both* Dipterus *and* Dipnorhynchus; *these are thought to be related to the pineal organ. More derived lungfish lack this feature.*

0. One or more extra canals

1. No canals

**20. Canal for anterior cerebral vein**

*The canal for the anterior cerebral vein bifuricates within the cranial cavity in* Dipterus *and* Griphognathus whitei, *whereas it remains a single canal in* Chirodipterus wildungensis, Chirodipterus australis, *and* Holodipterus gogoensis ([Challands 2015](#_ENREF_2)). *This condition is unknown in Dipnorhynchus.*

0. Bifuricates within cranial cavity

1. Single canal in cranial cavity

**Part C. Supplementary References**

Campbell KSW, and Barwick RE. 1982. The neurocranium of the primitive dipnoan *Dipnorhynchus sussmilchi* (Etheridge). *Journal of Vertebrate Paleontology* 2:286-327.

Challands TJ. 2015. The cranial endocast of the Middle Devonian dipnoan *Dipterus valenciennesi* and a fossilised dipnoan otoconal mass. *Papers in Palaeontology* 1:289-317.

Chang MM. 1982. The braincase of *Youngolepis*, a Lower Devonian crossopterygian from Yunnan, south-western China PhD. University of Stockholm and Section of Palaeozoology, Swedish Museum of Natural History.

Clement AM, and Ahlberg PE. 2014. The first virtual cranial endocast of a lungfish (Sarcopterygii: Dipnoi). *PloS One* 9:19. 10.1371/journal.pone.0113898

Clement AM, Nysjö J, Strand R, and Ahlberg PE. 2015. Brain – endocast relationship in the Australian lungfish, *Neoceratodus forsteri*, elucidated from tomographic data (Sarcopterygii: Dipnoi). *PloS One*. DOI: 10.1371/journal.pone.0141277

Friedman M. 2007. The interrelationships of Devonian lungfishes (Sarcopterygii: Dipnoi) as inferred from neurocranial evidence and new data from the genus *Soederberghia* Lehman, 1959. *Zoological Journal of the Linnean Society* 151:115-171.

Giles S, Friedman M, and Brazeau MD. 2015. Osteichthyan-like cranial conditions in an Early Devonian stem gnathostome. *Nature*. 10.1038/nature14065

Holland T. 2014. The endocranial anatomy of Gogonasus andrewsae Long, 1985 revealed through micro CT-scanning. *Earth and Environmental Science Transactions of the Royal Society of Edinburgh* 105:9-34.

Khorevin VI. 2008. The lagena (the third otolith endorgan in vertebrates). *Neurophysiology* 40:160-178.

Lu J, Zhu M, Ahlberg PE, Qiao T, Zhu Y, Zhao W, and Jia LT. 2016. A Devonian predatory fish provides insights into the early evolution of modern sarcopterygians. *Science Advances* 2:1-8.

Northcutt RG. 1986. Lungfish neural characters and their bearing on sarcopterygian phylogeny. *Journal of Morphology Supplement* 1:277-297.

Säve-Söderbergh G. 1952. On the skull of *Chirodipterus wildungensis* Gross, an Upper Devonian dipnoan from Wildungen. *Kunglinga Svenska Vetenskapsakademiens Handlingar 4* 3:1-29.

Stensiö E. 1963. The Brain and the Cranial Nerves in Fossil, Lower Craniate Vertebrates. *Skrifter utgitt av Det Norske Videnskaps-Akademi*:1-120.
