# Supplementary figures and images for: The cranial endocast of Dipnorhynchus sussmilchi (Sarcopterygii: Dipnoi) and the interrelationships of stem-group lungfishes
Source: PeerJ. 2016 Oct 20;4:e2539. doi: 10.7717/peerj.2539 (PMC5075708; doi:10.7717/peerj.2539)

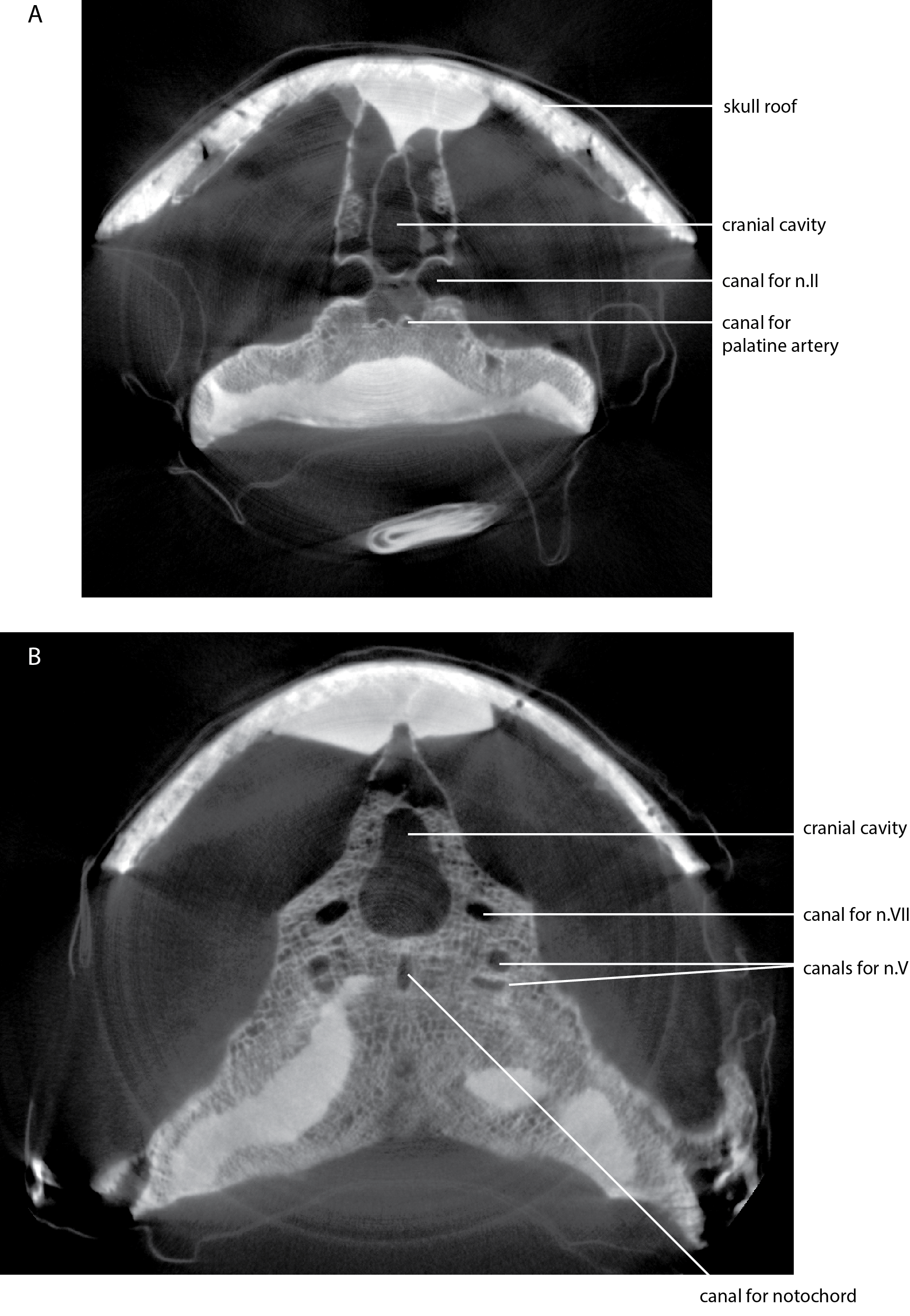

Supplement: Figure S1 [file peerj-04-2539-s002.png]
